# Searching the protein structure database for ligand-binding site similarities using CPASS v.2

**DOI:** 10.1186/1756-0500-4-17

**Published:** 2011-01-26

**Authors:** Robert Powers, Jennifer C Copeland, Jaime L Stark, Adam Caprez, Ashu Guru, David Swanson

**Affiliations:** 1 Department of Chemistry, University of Nebraska-Lincoln, Lincoln, NE 68588-0304 USA; 2 Holland Computing Center, University of Nebraska-Lincoln, Lincoln, NE 68588-0150 USA

## Abstract

**Background:**

A recent analysis of protein sequences deposited in the NCBI RefSeq database indicates that ~8.5 million protein sequences are encoded in prokaryotic and eukaryotic genomes, where ~30% are explicitly annotated as "hypothetical" or "uncharacterized" protein. Our Comparison of Protein Active-Site Structures (CPASS v.2) database and software compares the sequence and structural characteristics of experimentally determined ligand binding sites to infer a functional relationship in the absence of global sequence or structure similarity. CPASS is an important component of our Functional Annotation Screening Technology by NMR (FAST-NMR) protocol and has been successfully applied to aid the annotation of a number of proteins of unknown function.

**Findings:**

We report a major upgrade to our CPASS software and database that significantly improves its broad utility. CPASS v.2 is designed with a layered architecture to increase flexibility and portability that also enables job distribution over the Open Science Grid (OSG) to increase speed. Similarly, the CPASS interface was enhanced to provide more user flexibility in submitting a CPASS query. CPASS v.2 now allows for both automatic and manual definition of ligand-binding sites and permits pair-wise, one versus all, one versus list, or list versus list comparisons. Solvent accessible surface area, ligand root-mean square difference, and Cβ distances have been incorporated into the CPASS similarity function to improve the quality of the results. The CPASS database has also been updated.

**Conclusions:**

CPASS v.2 is more than an order of magnitude faster than the original implementation, and allows for multiple simultaneous job submissions. Similarly, the CPASS database of ligand-defined binding sites has increased in size by ~ 38%, dramatically increasing the likelihood of a positive search result. The modification to the CPASS similarity function is effective in reducing CPASS similarity scores for false positives by ~30%, while leaving true positives unaffected. Importantly, receiver operating characteristics (ROC) curves demonstrate the high correlation between CPASS similarity scores and an accurate functional assignment. As indicated by distribution curves, scores ≥ 30% infer a functional similarity. Software URL: http://cpass.unl.edu.

## Background

The Comparison of Protein Active-Site Structures (CPASS) [1] is an integral component of our FAST-NMR methodology [2,3] to annotate proteins of unknown function. CPASS is based on the premise that ligand-binding sites or functional epitopes are more evolutionary stable relative to the remainder of the protein [4,5]. Thus, a protein of unknown function is annotated by identifying proteins of known function that share similar ligand-binding sites [6]. The FAST-NMR and CPASS methodology is well-suited to situations where global sequence or structure similarity has failed to assign a function [7]. CPASS has contributed to a functional hypothesis for the *Staphylococcus aureus *protein SAV1430 [3], *Pseudomonas aeruginosa *protein PA1324 [8], *Pyrococcus horikoshii *OT3 protein PH1320 [2], and human protein Q13206 [2]. The basic CPASS approach has been used to provide an annotation to the *Bacillus subtilis *protein YndB [9]. Also, CPASS was used to identify a functional relationship between the bacterial type III secretion system and eukaryotic apoptosis [10].

CPASS compares the sequence and structural characteristics between *experimental *ligand-defined active-sites or functional epitopes to identify a functional relationship. This is uniquely different from other bioinformatic tools such as eF-seek [11], PINTS [12,13], ProFunc [14], and many others [15] that attempt to predict the location of ligand-binding sites based on structural features such as spatially conserved residues, surface pockets, or other physiochemical properties. Programs such as @TOME-2 [16], 3DLigandSite [17], and firestar [18] predict ligand binding sites or functional similarity through the global alignment of protein structures, where reference structures contain bound ligands. Conversely, ProteMiner-SSM [19], Query3d [20], and SiteBase [21] are similar in concept to CPASS, where only ligand-binding site substructures are used as a database query. CPASS uses the entire binding site defined from a direct interaction with a ligand, where any amino-acid that is within 6 Å of the bound ligand comprises the ligand-defined binding site. Thus, CPASS uses a comprehensive database comprised of every distinct ligand-binding site present in the RCSB Protein Data Bank (PDB) [22]. The presence of a different ligand, a global sequence similarity less than 90%, or an active site similarity less than 80% correlates with a unique binding site in the CPASS database. As a result, a CPASS search is extremely exhaustive, but time consuming. Conversely, other software that attempt to predict the location of a ligand-binding site typically use reduced definitions of known ligand binding sites, such as a triad of highly conserved residues. These approaches are optimized for speed, but generally identify numerous ambiguous ligand binding sites.

We report here a major upgrade to our CPASS software and database that significantly improves the broad utility of CPASS. The enhancements include more than an order of magnitude reduction in the time required to completely search the CPASS database, an approximate 38% increase in the size of the CPASS database of ligand binding sites, the incorporation of additional terms in our active-site similarity function that further differentiates true positives from false positives, and improvements in the CPASS user interface.

## Methods

### Prior CPASS implementation

As described in an earlier work [23], the CPASS suite previously utilized a 16-node Beowulf Linux cluster to both store the database and perform the computations. The various components of the suite (user interface (UI), preprocessing, computation, database, post processing) were tightly integrated, as well as non-portable. Additionally, the design was such that only one comparison sweep could be performed at a time, with no mechanism to queue jobs. This model served the purpose during the initial development, but had several inherent limitations for a larger user base.

The non-portable nature of the code (e.g. hard-coded file paths) meant that CPASS was not scalable beyond the original 16-node cluster. The single-user nature and lengthy computation time for a full comparison (~1 day) resulted in severely limited computational throughput. The tight integration of the components also limited flexibility for modification; a change in one area could require altering other components. Finally, there were no mechanisms for fault-tolerance in place. For example, a failure of one compute node, or in one layer of the application stack, could require the comparison to be begun again. Thus, there were several well-defined areas in which improvements could be made.

### Current CPASS implementation

**Figure 1:**
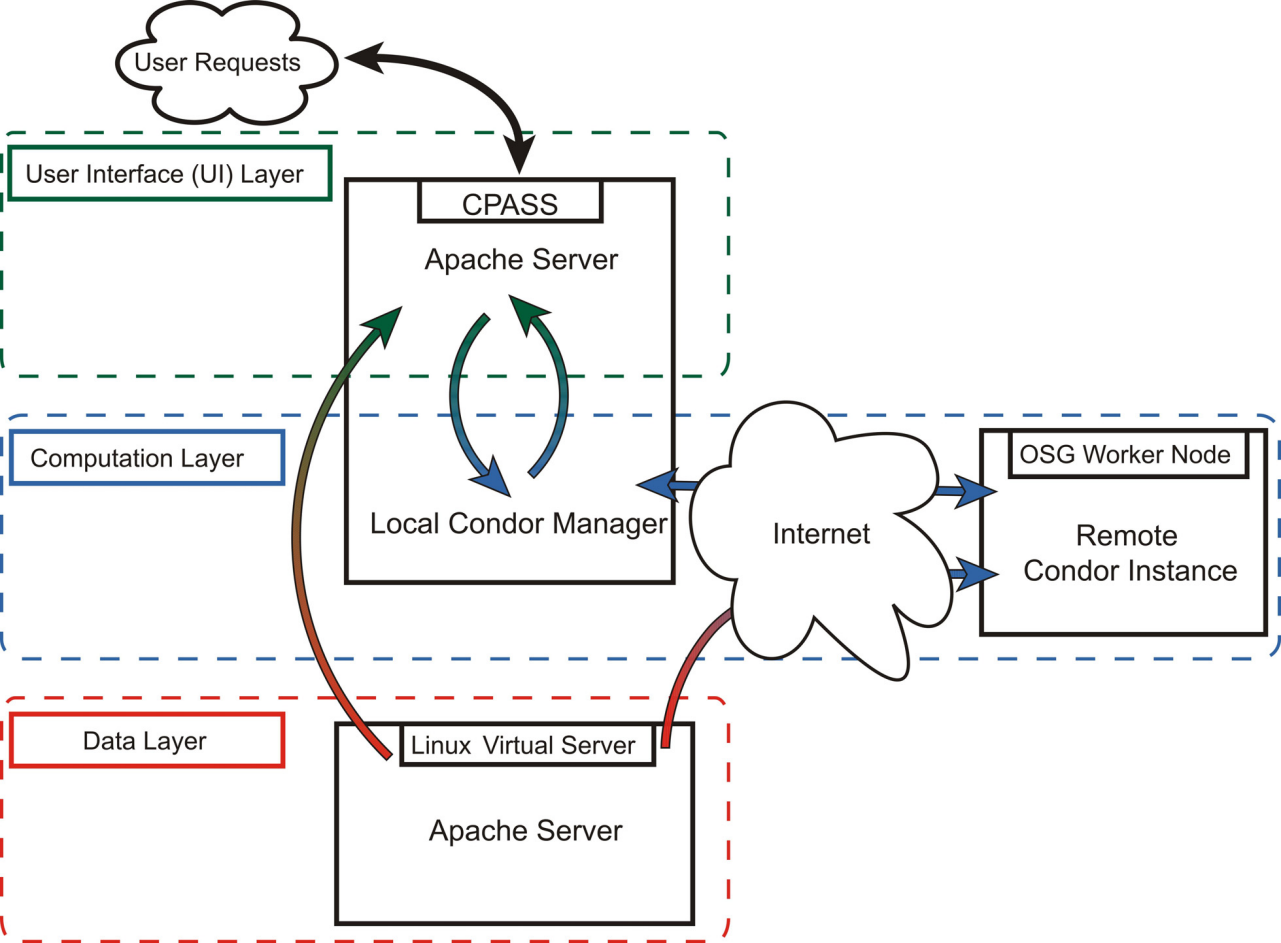
**Illustration of the three distinct layers in the current CPASS implementation. **The User Interface (UI) Layer processes user requests and displays results. The Computation Layer deploys jobs over the internet to remote Open Science Grid (OSG) worker nodes, and collects the results. The Data Layer provides necessary database files to both the UI and Computation Layers.

The current CPASS design directly addresses the issues described above. In order to allow scaling beyond the original cluster, all components were modified to be portable. Additionally, the previous tight integration has been replaced with a layered architecture (Figure 1). These consist of distinct UI, Computation, and Data layers, discussed below. The primary advantage of this approach is flexibility; each layer operates asynchronously, and may be modified independent of the others. Communication between the UI and Computation layers is file-based, with checkpoints present in the workflow. A degree of fault-tolerance is thus introduced; if either layer fails, loss is limited to a single layer, and the workflow can be resumed from the last checkpoint.

The UI layer runs on a single server, and is responsible for the user-facing web portal, pre-processing of the user-supplied data, and display of the results. When a user submits a CPASS comparison through the portal, the UI layer performs the pre-processing and logs the job details. Periodically, the logs are scanned for pending jobs, which are then sent to the Computation layer and logged as active. The Computation layer is monitored for job status, and upon completion, post-processing and logging are performed. The UI layer parses the log files, as well as directly querying the Computation layer, on demand from the user. If the job is complete, the results may be displayed. In the prior CPASS implementation, each individual comparison generated results as static html documents. Result pages are now generated dynamically from plain-text files produced at the Computation layer. Additionally, the current architecture allows multiple concurrent users. Upon submission, each comparison is assigned a unique working directory where all related data is kept. This allows multiple users to submit an arbitrary number of jobs, which are then queued at the Computation layer in the order they are received.

The Computation layer is responsible for executing the core CPASS functionality. A full CPASS sweep is broken into a cluster of several hundred independent jobs prior to submission to the Condor batch system [24,25], which is used in conjunction with glideinWMS [26]. Condor is a specialized workload management system designed for high-throughput computing. It is responsible for job queuing, scheduling, prioritization, resource monitoring, and resource management. The glideinWMS mechanism simplifies utilization of the hundreds of independent sites that form the Open Science Grid [27] (OSG). The combination of the two provides a scalable architecture for opportunistic use of Grid compute resources.

A Condor instance is run on the same server the UI layer resides on. The cluster of jobs is submitted to the local Condor instance, which is then distributed using glideinWMS to available Grid compute nodes for execution. Each job is responsible for comparison against a subset of the complete database. Upon completion of each job, the results are transferred back to its local working directory to be displayed by the UI layer. Utilizing the Condor scheduler also provides tolerance against job failure. As each individual job in the cluster is independent, any number may fail at any time without affecting the rest. Condor will detect the failure, and reschedule the job for execution at a different location. With this implementation jobs are run at disparate sites, thus a shared file system is no longer present. Consequently, the relevant database files must be distributed to the remote worker nodes, necessitating the addition of the Data layer.

The Data layer's role is to host the CPASS database, and serve the required files to individual jobs on demand. Given that the database itself consists of a large number of relatively small (approximately 1 MB) files, http-based distribution was chosen. The Data layer consists of a Linux Virtual Server [28] (LVS) instance, using the Apache HTTP Server for distribution. The LVS is a scalable, high availability (HA) server composed of a cluster of real servers with a Linux-based load balancer. Currently this consists of the required two load balancers (a primary and a backup), and two Apache web servers. As CPASS usage grows, this will place increasing demands on the Data layer to deliver files. The scalable nature of an LVS means that additional web servers may be added transparently to cope with increased demand. Additionally, the HA feature is such that one load balancer, and all but one web server, may fail without interruption to the overall LVS. The Computation layer can continue to operate, although possibly at a reduced level of performance depending on demand.

The current CPASS implementation addresses several core issues identified previously, with significant improvements in flexibility, scalability, and fault-tolerance. Separation of the architecture into distinct layers allows for ease of development. Portability and scalability ensures that as demand grows, additional resources may be utilized more easily. The addition of fault-tolerance in all levels aims to improve the user experience. Most importantly, the ability for high computational throughput will greatly enhance the value of CPASS as an analysis tool.

### CPASS similarity function

The original CPASS similarity function was based on a Cα distance-weighted BLOSUM62 [29,30] probability function.

(1) $$\begin{array}{cc} S_{ab}=\sum_{i,j=1}^{i=n,j=m}\frac{d_{min}}{d_{i}}{\left(e^{-\Delta \;RMSD_{i,j}}\right)}^{2}p_{i,j} & \Delta \;RMSD_{i,j}=\{\begin{array}{ll} RMSD_{i,j}-1 & RMSD_{i,j}>1\text{Å}\\ 0 & RMSD_{i,j}\leq 1\text{Å} \end{array} \end{array}$$

where active site *a *contains *n *residues and is compared to active site *b *from the CPASS database which contains *m *residues, *p*_*i,j *_is the BLOSUM62 probability for amino-acid replacement for residue *i *from active site *a *with residue *j *from active site *b*, *ΔRMSD*_*i,j *_is a corrected root-mean square difference in the Cα coordinate positions between residues *i *and *j*, and *d*_*min*_*/d*_*i *_is the ratio of the shortest distance to the ligand among all amino-acids in the active site compared to the current amino-acid's shortest distance to the ligand.

**Figure 2:**
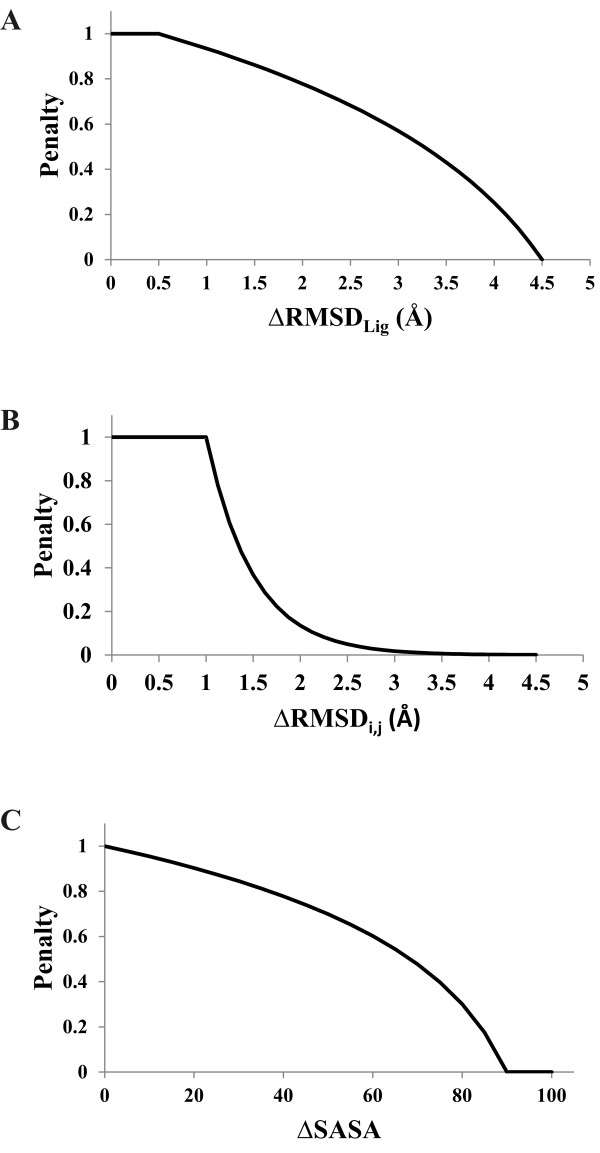
**Graphical representations of the penalty functions for (A) ligand root-mean square difference (RMSD), (B) per residue RMSD in Cα and Cβ positions, and (C) solvent accessible surface area (SASA) differences in the CPASS similarity score**.

The upgraded version of CPASS has incorporated three changes into the similarity function. These changes correspond to the inclusion of the solvent accessible surface area of the aligned residues, the root-mean square difference (RMSD) between the two bound ligands, and the addition of the Cβ position in the distance calculation (*ΔRMSD*_*i,j*_). For residue alignments that involve at least one glycine residue, the per residue distance calculation only uses Cα coordinate positions between residues *i *and *j*.

(2) $$S_{ab}=\log_{5}(5-\Delta RMSD_{lig})\sum_{i,j=1}^{i=n,j=m}\frac{d_{\min}}{d_{i}}(\log_{10}(100-\Delta SASA_{i,j})-1){\left(e^{-\Delta \;RMSD_{i,j}}\right)}^{2}p_{i,j}$$

where *ΔRMSD*_*lig *_is a corrected root-mean square difference between the ligands that define the two binding sites and *ΔSASA*_*i,j *_is the difference in the solvent accessible surface area (SASA) between residues *i *and *j*. The penalty functions for the RMSD between the two ligands, the per residue Cα-Cβ position in the distance calculation, and the solvent accessible surface area of the aligned residues are illustrated in figure 2.

The solvent accessible surface area for each residue in each structure is calculated using the program NACCESS [31]. Specifically, the relative all atom solvent accessible surface area is used. All heteroatoms in the PDB are ignored in the NACCESS calculation, so the solvent accessible surface area corresponds to a ligand-free structure. For structures that contain bound peptides or nucleotides, the peptides or nucleotides were removed from the PDB prior to the NACCESS calculation.

The RMSD between the two ligands is calculated by using the shortest distance to each non-hydrogen atom to the smallest (lowest number of heavy atoms) of the two ligands. It is common for the two ligands in the comparison to be unique chemical entities with a different number of atoms. So, each atom in the smallest structure is used to calculate the RMSD, while all the "extra" atoms in the larger structure are ignored. Effectively, the RMSD is measured from the smaller ligand to an aligned substructure of the larger ligand. The calculated RMSD is then reduced by 0.5 Å to provide a non-penalty region to accommodate for experimental error.

### CPASS database update

When CPASS was originally developed, a total of ~34,000 X-ray and NMR structures were available from the RCSB PDB [22]. This led to a CPASS database composed of ~26,000 unique ligand-defined binding sites. A ligand is broadly defined as any small molecular-weight organic compound (co-factors, drugs, metabolites, substrates, etc) or small peptide, DNA or RNA strand consisting of thirteen or less residues. A unique ligand-defined binding site implies that two binding sites that share the same ligand have less than 90% global sequence similarity or less than 80% sequence similarity in the ligand binding site. Common buffers, detergents, salts and other small ligands are removed from the CPASS database. Since the original inception, the RCSB PDB has increased significantly and contains ~68,000 X-ray and NMR structures as of September 2010. This has led to a corresponding increase in the CPASS database, which now comprises ~36,000 unique ligand-defined binding sites. The resulting increase in the CPASS database improves the coverage of functional space and increases the likelihood that a match will be found between a functionally uncharacterized protein and the CPASS database.

### CPASS user interface enhancements

**Figure 3:**
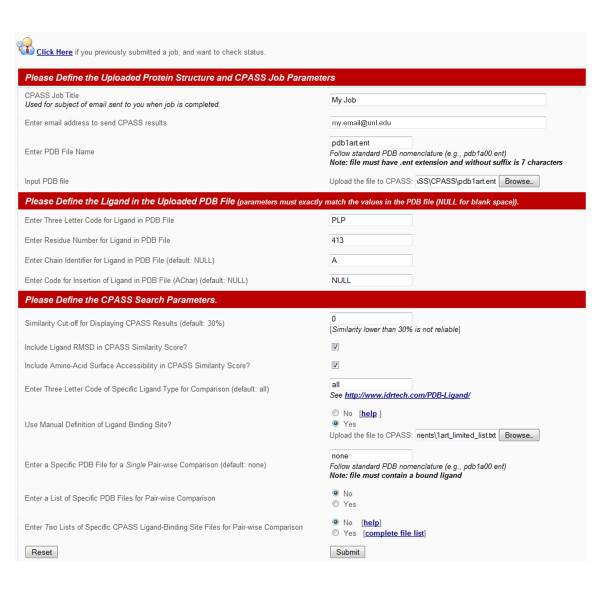
**An example of a completed CPASS submission page is shown that highlights the enhanced user flexibility in database selection and search criteria**. The queried ligand-defined binding site can be manually or automatically defined. The similarity search can be conducted against the entire CPASS database, against a single protein structure or against a user defined list of structures. The search can be further filtered by the type of ligand that defines the ligand binding site. Also, a similarity search can be conducted between two user defined lists of ligand-defined binding sites present in the CPASS database.

A number of valuable changes to the CPASS user interface have been incorporated to improve the ease of use of the program. The CPASS job submission page has been expanded to include more flexibility in how a CPASS calculation is performed (Figure 3). In the original version of CPASS, the user was limited to the comparison of a single protein target against the entire CPASS database. The CPASS database could be reduced to a subset of binding sites by defining a specific ligand (ATP, PLP, NADP, etc) defined binding site. These options were expanded in CPASS v.2, a single pair-wise comparison can be performed against a user-selected RCSB PDB structure. Similarly, a comparison can be calculated using a user-selected list of RCSB PDB structures. In both cases, the RCSB PDB structures must contain a bound ligand for a successful CPASS calculation. Also, the user-defined lists can be further filtered by defining a specific ligand (ATP, PLP, NADP, etc). Finally, the user can submit two lists of specific CPASS ligand-binding site files for a series of pair-wise comparisons. Choosing this option does not require the user to upload a protein-ligand structure to CPASS. Instead, the comparison is strictly between CPASS database files culled from the RCSB PDB. This requires the user to use the CPASS file nomenclature for defining a ligand-defined binding site. A file containing the complete, unfiltered list of ~100,000 CPASS ligand-defined binding sites is provided to the user to generate the two lists of specific CPASS ligand-binding site files for the pair-wise comparisons.

Furthermore, the original version of CPASS limited the ligand-defined binding site comparisons to an experimental protein-ligand co-structure uploaded by the user, where CPASS extracted the ligand-defined binding site based on the presence of a ligand in the uploaded structure. CPASS v.2 allows the flexibility of a manually defined ligand binding site, when an experimental protein-ligand co-structure is not available. The user simply provides a standard text file listing the residues in the uploaded protein structure that correspond to the predicted ligand binding site.

**Figure 4:**
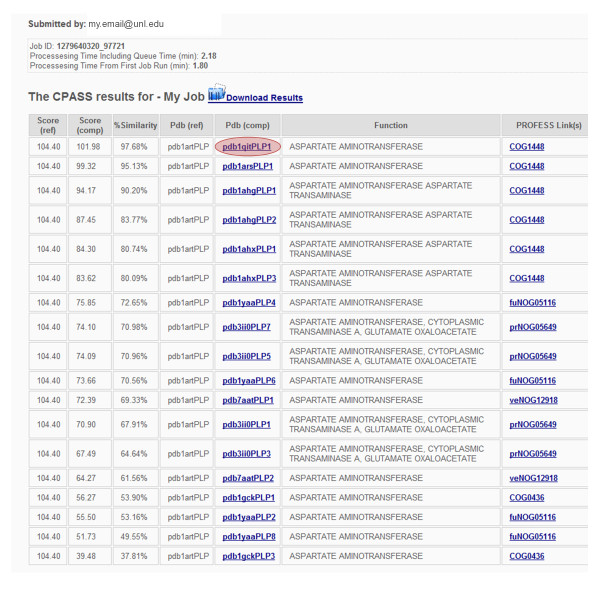
**An illustration of an overall CPASS results table that lists the reference (queried) CPASS score, the comparison CPASS score, the percent similarity between the two CPASS scores, and the CPASS IDs for the reference and comparison ligand-defined binding sites. **A functional description and a link to the corresponding PROFESS [25] page for each comparison protein is also provided. The CPASS ID (highlighted) for the comparison ligand-defined binding site is a link to a detailed description of the specific ligand-binding site alignment (see Figure 5).

The overall CPASS results table that lists all the ligand-defined binding sites with a similarity (usually > 30%) to the searched binding site has also been modified (Figure 4). Specifically, the results table now contains a column that contains a description of the function associated with each matched ligand-defined binding site. An additional column was also added that lists the eggNOG (http://eggnog.embl.de/) functional classification identification number [32], providing a direct link to a summary page in our PROtein Function, Evolution, Structure and Sequence (PROFESS) database (http://cse.unl.edu/~profess/) [33]. In this manner, it is now trivial to ascertain if a prevalent functional classification is apparent from a CPASS analysis.

**Figure 5:**
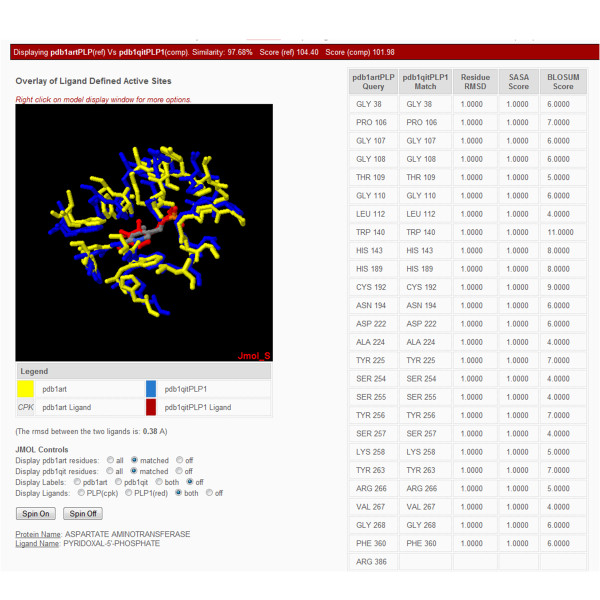
**A CPASS alignment summary page is shown that provides a detailed description of the specific ligand-binding site alignment. **An interactive Jmol [26] representation of the two aligned ligand defined active sites determined by CPASS is displayed. The RMSD between the two ligands, the protein name and ligand name for the comparison ligand-defined binding site are also provided. The associated table identifies the aligned residues and the per residue *ΔRMSD*_*i,j *_and *ΔSASA*_*i,j *_penalties, and the resulting weighted per residue BLOSUM62 score.

The detailed display of each CPASS aligned ligand-defined binding-site now includes the ligand structures (Figure 5). The calculated RMSD between the two ligands is displayed below the aligned structures. The program Jmol [34] (http://www.jmol.org/) has replaced Chime [35] for the graphical display of the overlap of the two aligned ligand-binding-site structures. Jmol is a Java-based molecular viewer that is actively being developed and significantly improves upon the capabilities of Chime. Jmol button options allow for the easy display of all the residues that define each ligand defined binding site, the display of only the aligned or matched residues between the two binding sites, or selectively turning off the display of either of the two aligned binding sites. Similarly, Jmol buttons can be selected to turn on or off residue labels or the ligand structure. The associated table lists the aligned residues between the two ligand-defined binding sites, the corresponding per residue *ΔRMSD*_*i,j *_and *ΔSASA*_*i,j *_penalties, and the resulting weighted per residue BLOSUM62 score.

### Evaluation of CPASS performance

Six different proteins were evaluated using CPASS: glycine hydroxymethyltransferase (PDB: 1 kkp, E.C. 2.1.2.1); aspartate transaminase (PDB: 1yaa, E.C. 2.6.1.1); pyruvate kinase (PDB: 3hqo, E.C. 2.7.1.40); phosphoenolpyruvate carboxykinase (PDB: 1xkv, E.C. 4.1.1.49); glutamine-tRNA ligase (PDB: 1gtr, E.C. 6.1.1.18); and biotin carboxylase (PDB: 1dv2, E.C. 6.3.4.14). The ATP or pyridoxal-5'-phosphate (PLP) ligand binding site from each protein structure was compared against the entire CPASS database of ~36,000 ligand-defined binding sites. Each of these query proteins was submitted using three different CPASS search parameters. A default CPASS search utilizes a ligand-defined binding site from an experimental NMR or X-ray co-structure with the additions to the similarity function of the ligand RMSD, solvent accessible surface area, and the Cβ position within the distance calculation. The two other searches either excluded the ligand RMSD, Cβ in the RMSD calculation, and solvent accessible surface area from the similarity function or used a manually-defined ligand binding site.

Three different methods were used to define what constitutes a functionally similar active site (true positive). The first method only used proteins that were assigned to the same Enzyme Commission (E.C.) classification [36] (i.e., all four E.C. numbers are identical), where the ligand-defined binding sites contained either the same ligand or a very similar ligand. The second method simply used a broader definition of E.C. similarity (i.e., only the first three E.C. numbers are required to be identical). The third method used a very broad definition of functional homology by defining all active sites in the database that bind the same ligand as being functionally similar. ROC curves were generated using the three different definitions of a true positive. The true positive rates were plotted against false positive rates over the full range of CPASS similarity scores using the different CPASS search parameters and the different definitions of true positives. Similarly, distribution curves plot the fraction of negatives and the fraction of positives at each CPASS similarity score using a bin size of 10. The fraction simply corresponds to the number of positives or negatives per bin relative to the total number of positives of negatives. The area under each curve is 1.

## Results

### Improvement in CPASS search speed

A notable limitation in the original implementation of CPASS was the significant time required to complete a search against the entire CPASS database. On average, a single comparison took ~40 s, requiring ~24 hrs to complete a search on our 16-node Beowulf Linux cluster. Obviously, the search time increased proportionally with the growth in the RCSB PDB database and the resulting CPASS database. This necessitated strict control over user access to prevent overwhelming our laboratory computer resources. In the recent upgrade, the CPASS code has been optimized, reducing a single comparison to ~7 s, which is greater than 5-fold improvement. CPASS has also been further modified to take advantage of resources available on the Open Science Grid. As a result, the CPASS calculation time has been reduced to less than an hour (including set-up time), more than an order of magnitude improvement. Importantly, this significant reduction in the CPASS search time enabled us to remove any user restrictions to routine access to CPASS. Furthermore, the dramatic improvement in speed is expected to greatly improve the wide-spread utilization of CPASS. CPASS is freely accessible to academic users through our web-site (http://cpass.unl.edu).

### CPASS similarity function

The overall philosophy behind the development of the CPASS database and program is the application of experimental ligand-defined binding sites to infer a functional annotation when global sequence and structure similarity is inconclusive. In this manner, CPASS attempts to quantify the structural and sequence similarity between two ligand defined binding sites by spatially overlaying similar residue types. CPASS was primarily designed to compare *experimental *ligand-defined binding sites. Unfortunately, a protein-ligand co-structure is not always available, but in some cases the identity of the ligand-binding site may be inferred from other sources, such as site-directed mutagenesis, NMR chemical shift perturbations, bioinformatics, or computer modeling.

The new version of CPASS allows for the manual identification of the ligand binding site in addition to the typical extraction of the ligand-binding site from an uploaded protein-ligand PDB file. The manual definition of the ligand-binding site simply requires uploading a standard text file to CPASS. The text file should list the three letter amino acid abbreviation for each residue in the binding site, and the corresponding residue number and chain identifier. The information should exactly match the corresponding residue identifiers in the protein PDB file that is also uploaded to CPASS. The use of a manually-defined ligand-binding site also requires a subtle change in the CPASS similarity function (see eqn. 2), since the structure does not contain a bound ligand. First, the aligned ligand RMSD penalty function is disabled. Second, the ratio of the shortest distance to the ligand (*d*_*min*_) among all amino-acids in the active site compared to each amino-acid's shortest distance to the ligand (*d*_*i*_) requires a new reference point since the ligand is not present. The ligand reference point is simply replaced by the center-of-mass for the manually defined binding site. This scaling factor simply reduces the contribution of residues at the 6 Å edge for inclusion in the ligand binding site definition. It diminishes the impact of small structural variations that may result in either the inclusion or exclusion of residues at the 6 Å limit that would correlate to an unjustified large difference in the CPASS similarity score.

CPASS uses a distance-weighted BLOSUM62 scoring function (see eqn. 2) to align and rank ligand-defined binding sites. The alignment ignores sequential connectivity and primarily focuses on the relative spatial orientations of the residues that comprise each binding site. Importantly, the identity or conformation of the ligand is not used in this alignment process. To further improve the ability of the CPASS similarity functions to eliminate dissimilar ligand-binding sites, three additions to the CPASS scoring function have been implemented.

CPASS uses the bound ligands to define the binding sites or functional epitopes, but the ligands are not used in the alignment process. This provides an additional mechanism to evaluate and rank the aligned ligand-binding sites, since the same transformation that was applied to align the binding sites are equally applied to each ligand. Thus, a binding site alignment that also results in a close alignment between the two bound ligands, especially if similar functional groups overlap, increases the likelihood that the two aligned proteins share a common function. The ligand alignment function (*ΔRMSD*_*lig*_) was empirically designed to gradually apply an increasing penalty as the RMSD between the two ligands increases; reaching a value of zero when the ligands are separated by > 4.5 Å (Figure 2A). The ligand alignment function does not provide a penalty when the aligned ligands are within 0.5 Å. This compensates for the typical experimental error encountered when comparing similar protein structures. A conservative function is applied since the ligand alignment is a global parameter that simply scales the CPASS similarity score. A large penalty based on a poor ligand alignment effectively defines the two ligand-binding sites as dissimilar regardless of how well the ligand-binding sites are aligned. CPASS also provides an option to exclude the ligand alignment function from the overall similarity score.

The original CPASS spatial alignment of ligand binding sites was based on Cα distances. This clearly captures the backbone orientations, which is the primary structural factor that determines ligand-binding site similarities, but it does ignore subtle and potentially important differences in side chain orientations. This issue was reduced by also including Cβ distances in the per residue distance alignment (*ΔRMSD*_*i,j*_) calculation in the new version of CPASS, requiring a corresponding upgrade to the CPASS database file structure. Of course, only Cα distances are used for alignments involving a glycine. The per-residue distance alignment function (Figure 2B) does not provide a penalty when the aligned residues are within 1 Å. This compensates for the typical experimental error encountered when comparing similar protein structures. But, the function decays rapidly as the RMSD increases beyond 1 Å, where a residue's alignment makes an insignificant contribution to the overall similarity score when the RMSD is greater than 2.5 Å. A relatively harsh per-residue penalty is warranted since the overall similarity score is based on the sum of all the aligned residues. Basically, two ligand binding sites that share an average per residue RMSD of greater than 2.5 Å are not very similar. As a comparison, consider the fact that highly similar protein structures have a global RMSD of less than 2.5 Å [37].

The per-residue solvent accessible surface area (SASA) captures a distinct physical descriptor that is unique from both the residue identity and the distances between aligned residues and bound ligands. Presumably, the overall characteristics of functionally related ligand binding sites, including SASA, should be preserved. Specifically, a shallow ligand binding cleft on the protein's surface is distinct from a ligand binding pocket formed at the interface of two proteins or domains, or from a deep-binding pocket where a majority of the binding site residues are buried below the protein's surface. Dissimilarity in SASA would further discriminate between these ligand binding sites even if there is a serendipitous spatial overlap in a sub-set of residues. The SASA function (Figure 2C) was empirically designed to emphasize the penalty for large SASA differences (≥60) that primarily distinguishes between surface accessible and buried residues. Similar to the ligand alignment function, the user also has the option to exclude the SASA difference in the CPASS similarity score.

**Figure 6:**
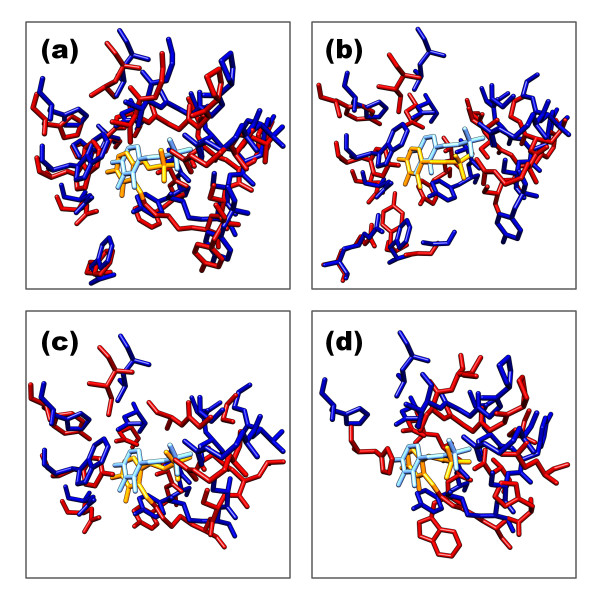
**Illustrations of CPASS similarity scores using a representative aspartate aminotransferase protein (PDB ID 1art) and four different pyridoxal-5'-phosphate (PLP) dependent proteins: (a) aspartate aminotransferase (PDB ID 1asf) with a CPASS similarity of 82.73%, (b) LL-diaminopimelate aminotransferase (PDB ID 3ei8) with a CPASS similarity of 50.09%, (c) histidinol-phosphate aminotransferase (PDB ID 1gew) with a CPASS similarity of 42.13%, and (d) cysteine sulfinic acid decarboxylase (PDB ID 2jis) with a CPASS similarity of 30.85%, at the low end of the confidence limit for a functional relationship**. The ligand defined binding sites are shown as licorice bonds where the molecular graphics images were produced using the UCSF Chimera (http://www.cgl.ucsf.edu/chimera) package from the Resource for Biocomputing, Visualization, and Informatics at the University of California, San Francisco [52]. The aspartate aminotransferase protein (PDB ID 1art) is colored blue in each panel, with PLP colored light blue. The other four PLP-dependent proteins in the binding site comparisons are colored red and the bound PLP is colored orange.

The various CPASS similarity functions were empirically designed to proportionally emphasize the contributions of residue identity, per residue alignment, SASA and ligand alignment to the overall sequence and structural similarity between two ligand binding sites. Thus, the CPASS similarity score that indicates two ligand binding sites are similar is also empirically evaluated. In general, CPASS similarities score greater than 30% suggests a moderate level of similarity between two ligand binding sites. The quality and the confidence in the ligand-binding site similarity increase with the CPASS similarity score. This point is easily illustrated by the comparison between the aspartate aminotransferase protein (PDB ID 1art) PLP binding site with four different PLP-dependent proteins, where the CPASS similarity score decreases from ~80% to ~30% (Figure 6).

### Evaluation of CPASS performance

The definition of a true positive is essential to the evaluation of CPASS performance. Ideally, a measurement of functional similarity would provide the necessary framework to define a true positive, but functional homology is still extremely challenging to quantitate [38]. There are several methods for functional classifications based on sequence similarity (COG [39], eggNOG [40], OMA [41]), structure similarity (CATH [42], SCOP [43]), or annotations (Gene Ontology, GO). Unfortunately, there are significant errors associated with each approach. GO terms are generally reliable and are the current "gold standard", but the annotations are often incomplete and overly generic. Functional clustering using sequence similarity may be too coarse (COG), which results in the inclusion of paralogs [39,44]; or too fine (eggNOG, OMA), which results in multiple clusters with the same function [45]. Of course, there are numerous examples of proteins that share the same function, but exhibit minimal sequence similarity [46,47]. Alternatively, functional divergence increases significantly as sequence identity drops below 50% [48]. Similar issues arise with structure similarities; there are proteins that exhibit the same function but have different structures, as well as the reverse [49-51]. Thus, the Enzyme Commission (E.C.) number was the best approach to define the functional similarity between the six query proteins and proteins within the CPASS database. E.C. numbers classify proteins based on enzyme-catalyzed reactions, which provides a generally reliable, but limited, mechanism to infer homologous functions.

**Figure 7:**
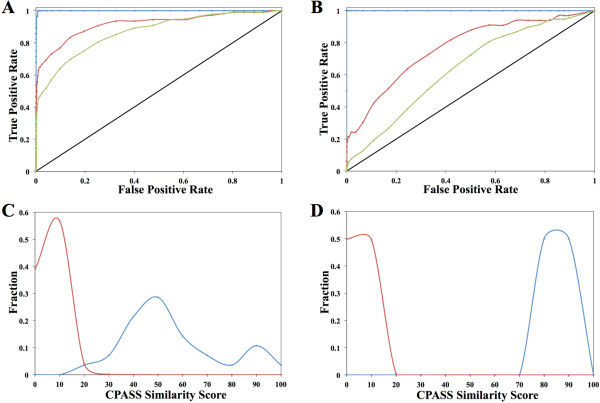
**ROC curves showing the true positive rate relative to the false positive rate of CPASS calculations for (A) aspartate transaminases (EC 2.6.1.1) and (B) glutamine-tRNA ligases (EC 6.1.1.18)**. True positives are defined based on three levels of functional homology between the query protein and the CPASS database. ROC curve with true positives based on an exact EC classification (all four E.C. numbers are identical) is colored blue. ROC curve with true positives based on a broad EC classification (only first three E.C. numbers are identical) is colored red. ROC curve with true positives based simply on proteins binding the same ligand is colored green. Distribution curves showing the fraction of negatives (red) and positives (blue) as a function of CPASS similarity scores (bin size of 10) are shown for (C) aspartate transaminases (EC 2.6.1.1) and (D) glutamine-tRNA ligases (EC 6.1.1.18).

Receiver operating characteristics (ROC) curves for two representative CPASS calculations of the proteins, aspartate transaminase (PDB: 1yaa, E.C. 2.6.1.1) and glutamine-tRNA ligase (PDB: 1gtr, E.C. 6.1.1.18) are shown in figure 7A,B. The ROC curves illustrate the overall ability of CPASS to identify true positives relative to false positives. The straight-line in the graph indicates the expected results if the CPASS predictions were completely random. The performance improves as the curve moves to the upper-left. As apparent in figure 7A,B, the enrichment in the ROC curves and the corresponding improvement in CPASS performance follows the increasing accuracy in the functional classification of true positives. The ROC curve where true positives are based on identical E.C. numbers is essentially ideal. The ROC curve based on a broader E.C. similarity (only the first three numbers are identical) is, as expected, reduced relative to the ROC curve based on identical E.C. numbers. But, this still shows an improvement over the ROC curve using true positives based only on proteins binding the same ligand. These results are not surprising. An increase in the accuracy of defining a functional similarity minimizes the number of proteins with low CPASS scores that are incorrectly characterized as true positives. It is important to note that in all cases the number of true positives based on identical E.C. numbers for each of the six query proteins is extremely small (2-28) relative to the size of the CPASS library (~36,000). This occurs because the CPASS library has been purposively filtered to remove identical or highly similar ligand-binding sites. The high CPASS performance is also illustrated by the distribution of the true positives and true negatives as a function of CPASS scores. The CPASS analysis of aspartate transaminase (Figure 7C) and glutamine-tRNA ligase (Figure 7D) indicate that true negatives peak at a CPASS score of ~10%. Conversely, true positives have a range of CPASS scores, but a threshold of ~20-30% is expected to identify the majority of functionally homologous proteins, while nearly eliminating false positives. Similar results were obtained for all six query proteins.

The impact on CPASS performance by the addition of ligand RMSD, Cβ in the RMSD calculation, and SASA to the similarity function was also evaluated. Similarly, the manual definition of a ligand binding site was compared to the experimental definition of a ligand binding site from an NMR or X-ray structure. The default CPASS approach uses the ligand of a protein-ligand co-structure to define the ligand binding site. As previously discussed, the default definition of a ligand-defined binding site utilizes a scaling function in the CPASS similarity score, where residues further from the ligand contribute less to the overall score (see eqn. 2). Since a manually defined ligand binding site lacks a ligand, the scaling factor uses distances from the center-of-mass for the manually defined binding site. A ROC curve analysis comparing the ligand-defined and manually-defined binding sites for a CPASS calculation with aspartate transaminase shows no significant difference in CPASS performance (data not shown). A similar result was obtained when comparing a CPASS calculation with or without the inclusion of Cβ in the RMSD calculation, ligand RMSD and SASA in the CPASS similarity function. Again, similar results were obtained for all six query proteins.

**Figure 8:**
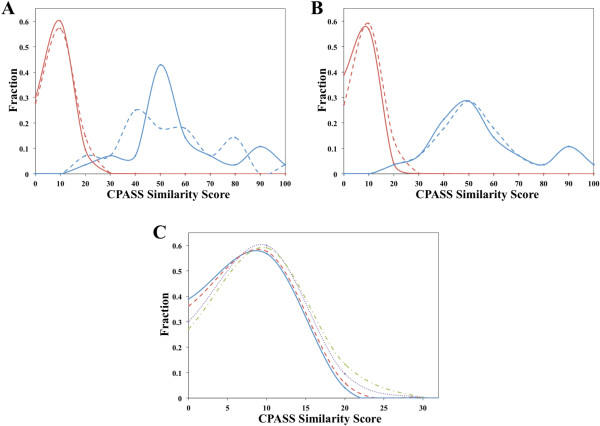
**Representative distribution curves showing the fraction of negatives and positives as a function of CPASS similarity scores for aspartate transaminases (PDB: 1yaa, EC 2.6.1.1)**. (A) A comparison of negatives (red) and positives (blue) CPASS similarity score distributions using a ligand-defined binding site (solid) and a manually-defined binding site (dashed). (B) A comparison of the fraction of negatives (red) and positives (blue) over the range of similarity scores for CPASS v.2, which includes the addition of Cβ in the RMSD calculation, ligand RMSD, and solvent accessible surface area (solid) to the original implementation of CPASS that lack these features (dashed). (C) A comparison of the fraction of negatives over the range of similarity scores for CPASS v.2 with all features (solid blue), CPASS v.2 without the addition of Cβ in the RMSD calculation (dashed red), CPASS v.2 without ligand RMSD and solvent accessible surface area (dotted purple), and CPASS v.1 (dash-dot green).

The similar performance using a manually-defined binding site demonstrates the relative robustness of the CPASS method. This is also evident by the clear distinction between true positives and true negatives seen in figure 7C,D. Nevertheless, the ROC curves do not capture the subtle differences in the CPASS results caused by using a manually-defined binding site. Figure 8A illustrates a typical distribution in CPASS similarity scores. The relative separation between the maximal peaks for negatives and positives is diminished for the manually-defined binding site. The CPASS scores for negatives increase and the scores for positives decrease, resulting in an increase in the overlap at the typical 20-30% threshold. Also, there is a wider range in the CPASS similarity scores for the positives. Despite these changes in the CPASS score distribution, the ROC curves are essentially identical indicating that the rate of identifying true positives relative to false positives is maintained. Thus, a manually-defined binding site still provides very reliable CPASS results, but the difference in similarity scores between positives and negatives is diminished relative to a ligand-defined binding site.

Similarly, the lack of an apparent improvement in the ROC curves by the inclusion of the ligand RMSD, the Cβ in the RMSD calculation, and SASA in the CPASS similarity function is not surprising given the nearly ideal performance of CPASS seen in figure 7A,B. Instead, the new CPASS similarity function was primarily expected to reduce the similarity score for negatives, while leaving positive scores unaffected. Effectively, the improvements to the CPASS similarity function were anticipated to enhance the differentiation between positives and negatives. A representative distribution of CPASS similarity scores comparing CPASS v.1 and CPASS v.2 is shown in figure 8B. As expected, the distribution of positive scores is basically unchanged. Similar ligand-defined binding sites are expected to have essentially identical side-chain orientations, per residue solvent accessible surface areas, and ligand conformations. Conversely, the CPASS similarity scores decrease for negatives because of an apparent deviation in these structural parameters. This is further illustrated in figure 8C by the sequential decrease in the fraction of negatives with scores above 20% as the new structural features are incrementally added to the CPASS similarity function. A threshold of 20-30% in the CPASS similarity score is typically used to identify potential functional homologs. Thus, the new CPASS similarity function is more efficient at eliminating false positives near this threshold. This is potentially very critical for the analysis of uncharacterized proteins, where a higher confidence in identifying a functional homolog is achieved even with a modest CPASS similarity score (≥ 30%).

## Conclusion

The overall goal of the CPASS database and software is to identify similar experimentally-determined ligand binding sites through an exhaustive pair-wise search of the RCSB PDB. CPASS optimizes the spatial orientation of similar amino-acids between two ligand-defined binding sites and ranks the alignment using a collection of sequence and structural empirical functions. We report a series of significant upgrades in CPASS v.2 that includes a dramatic improvement in speed, an expansion in the CPASS database of ligand defined binding sites, and modifications to the CPASS similarity scoring function and user interface.

## Competing interests

The authors declare that they have no competing interests.

## Authors' contributions

RP conceived and wrote the CPASS program and database, and participated in its design and coordination and drafted the manuscript. AC, AG and DS carried out the implementation of the current layered CPASS architecture and its use of the Open Science Grid, and contributed to the manuscript. JC and JS participated in the design of CPASS, were responsible for validating and testing the software and contributed to the manuscript. All authors read and approved the final manuscript.
